# Neurocomputational Consequences of Evolutionary Connectivity Changes in Perisylvian Language Cortex

**DOI:** 10.1523/JNEUROSCI.2693-16.2017

**Published:** 2017-03-15

**Authors:** Malte R. Schomers, Max Garagnani, Friedemann Pulvermüller

**Affiliations:** ^1^Brain Language Laboratory, Freie Universität Berlin, 14195 Berlin, Germany,; ^2^Berlin School of Mind and Brain, Humboldt-Universität zu Berlin, 10099 Berlin, Germany,; ^3^Centre for Robotics and Neural Systems, University of Plymouth, Plymouth PL4 8AA, United Kingdom, and; ^4^Department of Computing, Goldsmiths, University of London, London SE14 6NW, United Kingdom

**Keywords:** action–perception cycle, arcuate fasciculus, cortical connectivity, neurocomputational modeling, perisylvian cortex, verbal working memory

## Abstract

The human brain sets itself apart from that of its primate relatives by specific neuroanatomical features, especially the strong linkage of left perisylvian language areas (frontal and temporal cortex) by way of the arcuate fasciculus (AF). AF connectivity has been shown to correlate with verbal working memory—a specifically human trait providing the foundation for language abilities—but a mechanistic explanation of any related causal link between anatomical structure and cognitive function is still missing. Here, we provide a possible explanation and link, by using neurocomputational simulations in neuroanatomically structured models of the perisylvian language cortex. We compare networks mimicking key features of cortical connectivity in monkeys and humans, specifically the presence of relatively stronger higher-order “jumping links” between nonadjacent perisylvian cortical areas in the latter, and demonstrate that the emergence of working memory for syllables and word forms is a functional consequence of this structural evolutionary change. We also show that a mere increase of learning time is not sufficient, but that this specific structural feature, which entails higher connectivity degree of relevant areas and shorter sensorimotor path length, is crucial. These results offer a better understanding of specifically human anatomical features underlying the language faculty and their evolutionary selection advantage.

**SIGNIFICANCE STATEMENT** Why do humans have superior language abilities compared to primates? Recently, a uniquely human neuroanatomical feature has been demonstrated in the strength of the arcuate fasciculus (AF), a fiber pathway interlinking the left-hemispheric language areas. Although AF anatomy has been related to linguistic skills, an explanation of how this fiber bundle may support language abilities is still missing. We use neuroanatomically structured computational models to investigate the consequences of evolutionary changes in language area connectivity and demonstrate that the human-specific higher connectivity degree and comparatively shorter sensorimotor path length implicated by the AF entail emergence of verbal working memory, a prerequisite for language learning. These results offer a better understanding of specifically human anatomical features for language and their evolutionary selection advantage.

## Introduction

One of the key questions about human nature addresses the brain mechanisms underlying the language faculty. In sharp contrast to their closest relatives, humans learn novel words effortlessly and extremely rapidly (Shtyrov et al., 2010; Kimppa et al., 2015) and build vocabularies of tens of thousands of words (Pinker, 1994; Brysbaert et al., 2016), which can also be stored in verbal working memory (VWM). We here ask which neural mechanisms and features of brain–structural connectivity might enable these uniquely human abilities.

**Figure 1.**
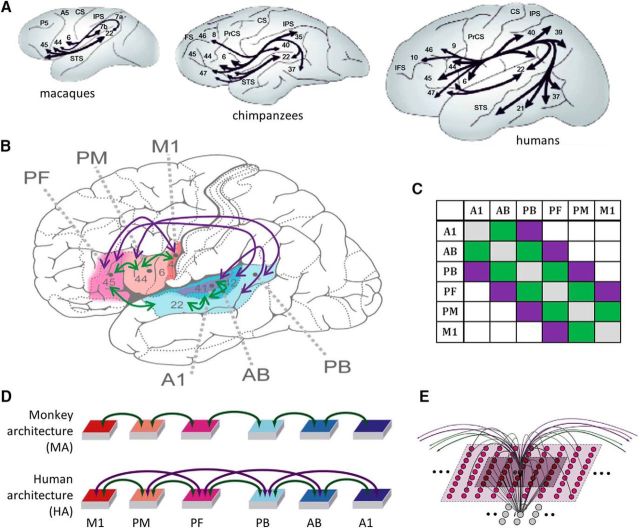
***A***, Illustration of perisylvian connectivity structure in macaques, chimpanzees, and humans as revealed by tractography studies [adapted by permission from Macmillan Publishers Ltd: Nature Neuroscience (Rilling et al., 2008), copyright 2008]. Note the strong frontotemporal connectivity of the latter, especially through the dorsal AF curving around the sylvian fissure, and the presence of ventral connections in both. ***B***, A human brain schematic is used to illustrate the area subdivision of the primate frontotemporal perisylvian cortex into M1, PM, and PF, and A1, AB, and PB areas (Garagnani et al., 2008). Green arrows give the connections available in both the human and monkey architecture (HA, MA); purple arrows give connections unique to the human architecture. The purple arrows present only in the HA are meant to reflect the additional connectivity strength available only in humans, as shown by comparative DTI/DWI studies (see main text for detailed discussion). ***C***, Connectivity matrix schematizing the connections according to next-neighbor (green) and indirect, jumping links (purple) skipping one intermediate area. ***D***, Schematic depiction of the neural network architectures, equivalent to ***B***. ***E***, Microstructure of the connectivity of one single excitatory cell (labeled “e”). Local (lateral) inhibition is implemented by an underlying cell “i” (representing a cluster of inhibitory interneurons situated within the same cortical column), which receives excitatory input from all cells situated within a local (5 × 5) neighborhood (dark-colored area) and projects back to e, inhibiting it. Within-area sparse excitatory links (in gray) to and from e are limited to a (19 × 19) neighborhood (light-colored area); between-area excitatory projections (green and purple arcs) are topographic and target 19 × 19 neighborhoods in other areas (not depicted). Panel ***B*** has been adapted from Garagnani and Pulvermüller (2013); panels ***D*** and ***E*** have been adapted from Cortex, 57, Pulvermüller, F. and Garagnani, M., “From sensorimotor learning to memory cells in prefrontal and temporal association cortex: A neurocomputational study of disembodiment”, pp. 1–21, copyright 2014, with permission from Elsevier.

Comparative neuroanatomical investigations using diffusion tensor imaging (DTI) and diffusion weighted imaging (DWI) along with invasive tracer studies in nonhuman primates have greatly advanced the search for the specific structural features of the human brain. Lesion evidence shows that inferior frontal [including Brodmann areas (BAs) 44/45] and superior temporal areas (BAs 42/22) of the left perisylvian cortex are most crucial for language, as lesions therein lead to aphasias involving both language production and comprehension (Bates et al., 2003). These core language areas are connected by a dorsal fiber bundle, the arcuate fasciculus (AF; Schüz and Braitenberg, 2002), providing a bidirectional link (Matsumoto et al., 2004). Whereas the ventral connections between these areas do not seem to have changed massively in primate evolution, this dorsal bundle via the AF is rich and strong in humans (Rilling et al., 2008), available already shortly after birth, and strongly lateralized to the left hemisphere (Dubois et al., 2009, 2014)—the language-dominant hemisphere in most people. Invasive tracing studies of macaque brains revealed a similar dorsal link between temporal parabelt and prefrontal areas (Petrides and Pandya, 2009), but parallel DTI/DWI and tractography in humans and macaques indicate relatively richer direct connections between inferior prefrontal and temporal parabelt areas in humans (Rilling et al., 2012). In addition to this quantitative statement, specific qualitative differences appear to be present within the AF, where some area-specific long-distance connections seem to have strengthened massively or may even have newly emerged in the evolution from macaque and chimpanzee to human. Whereas comparative neuroanatomical DTI studies show connections between prefrontal cortex and temporal areas in the auditory parabelt in both humans and monkeys (Thiebaut de Schotten et al., 2012, their Fig. 3), the additional links between prefrontal and auditory belt and between premotor and auditory parabelt areas are well documented with DTI/DWI in humans but not so in macaques or chimpanzees (Fig. 1*A*; Rilling et al., 2012; Thiebaut de Schotten et al., 2012); as these connections introduce shortcuts to what can be described as a 5-step next-neighbor architecture (Fig. 1*C*,*D*), we call them “jumping links.” Although not implying a complete absence of jumping links in nonhuman primates (Romanski et al., 1999; Smiley et al., 2007; Scott et al., 2015), the DTI-documented evolutionary change in dorsal connectivity leads to a shorter path length (defined as minimal number of synaptic steps) of strong links between auditory and articulatory motor areas. The AF appears crucially important for language, not only because of this evolutionary change, but also because its strength correlates with numerous human language abilities (Yeatman et al., 2011; López-Barroso et al., 2013; Saygin et al., 2013). However, a neuromechanistic explanation for why, among other factors, the quantitative topological differences in connectivity may be vital for the emergence of human-like language is still missing.

We here address this question using a novel approach of neurocomputational modeling, which has key advantages over both comparative studies and correlational evidence linking AF strength to language abilities. In those studies, a range of alternative features also distinguishing between monkey and human brains (including cortical area size and fiber diameters) could partly explain the observed performance differences. In contrast, models can be specifically designed to differ only in their connectivity structure, so that any functional change between them allows for definitive causal conclusions. We asked whether word learning or VWM abilities of humans could be causally linked to the presence of relatively stronger jumping links in human perisylvian cortex, as suggested by DTI/DWI data.

## Materials and Methods

### Network structure and function

We used a neurocomputational model of the perisylvian language cortex. These networks were composed of graded response cells thought to represent the average activity of a local pool of neurons (Eggert and Van Hemmen, 2000). Networks were subdivided into model areas of 25 × 25 = 625 excitatory and the same number of inhibitory neurons each (Fig. 1*E*). One model area was established for each of the following perisylvian areas (Fig. 1*B*; Garagnani et al., 2008): superior-temporal primary auditory cortex (A1), auditory belt (AB), and parabelt (PB) areas and inferior-frontal articulatory motor (M1), premotor (PM) and prefrontal (PF) cortex. Adjacent areas in all models were connected, based on reciprocal links documented between the corresponding brain areas [Fig. 1*B*, green-colored arrows (e.g., A1 to AB, AB to PB); Pandya and Yeterian, 1985; Braitenberg and Pulvermüller, 1992; Pandya, 1994; Young et al., 1994, 1995; Kaas and Hackett, 2000; Rauschecker and Tian, 2000].

As outlined in the Introduction, the rationale for this study was to investigate the functional consequence of qualitative and quantitative differences in connectivity between temporal and frontal regions along the dorsal stream. We therefore implemented two model architectures, a monkey architecture (MA) and human architecture (HA). In creating these architectures, we focused on major differences in the connectivity structure between monkey and human perisylvian regions that have been suggested by DTI/DWI-based tractography. This method currently offers the only prospect for comparative neuroanatomy of cortical long-distance connectivity, as invasive tracer studies are not possible in humans. We did not aim at modeling the full complexity of the connectivity structures in each species, because even tractography data of exceptionally high quality are not as accurate as neuroanatomical tracing data (Thomas et al., 2014) and therefore may not allow one to uncover all functionally relevant links in a given species. However, DTI/DWI tractography studies converge on showing stronger left frontotemporal connections in humans compared with nonhuman primates and more specifically the unique presence of strong jumping links (see Introduction). Therefore, we focus on modeling these differences between species, rather than complete architectures. Whereas the MA included only next-neighbor connections between adjacent areas, the HA included additional jumping links (Fig. 1*B–D*, purple). The strengths of all links were identical.

In addition to the between-area connectivity, which differed between the network architectures, both architectures were designed so as to mimic a range of biologically realistic properties and therefore included the following features: (1) within-area connectivity, which was random, sparse (thus realizing only a small fraction of all possible connections), patchy, and topographic (Gilbert and Wiesel, 1983; Amir et al., 1993; Braitenberg and Schüz, 1998), and such that local connection probability fell off with distance (Braitenberg and Schüz, 1998; Perin et al., 2011); (2) local and area-specific inhibition mechanisms (Fig. 1*E*, caption; Palm, 1982; Bibbig et al., 1995; Wennekers et al., 2006), which act as a means to regulate and control activity in the network (Braitenberg, 1978; Palm, 1982; Garagnani et al., 2008); (3) synaptic modification by way of Hebb-type learning including both long-term potentiation (LTP) and long-term depression (LTD; Artola et al., 1990; Artola and Singer, 1993); and (4) constant presence of uniform, uncorrelated white noise during all phases of learning and retrieval in all parts of the network (Rolls and Deco, 2010).

The implementation of the computational model follows that used in previous publications (Garagnani et al., 2008, 2009; Garagnani and Pulvermüller, 2013; Pulvermüller and Garagnani, 2014). Details about the underlying computations are also given in the section on Full model specification.

### Simulation procedures

Simulations consisted of the following two phases: the learning phase and the testing phase. Twelve pairs of network instances were built, with each pair consisting of one MA and one HA network (i.e., 24 networks in total). In each instance, we first initialized an HA network, which entailed (1) randomizing all synaptic links (and corresponding weights) between cells in neighboring areas (and within areas) and (2) randomly generating 14 sensorimotor patterns (“words”) to be used during training. Following this HA initialization, the network was copied, preserving the initial random links and the set of to-be-learned patterns, and the additional jumping links (Fig. 1*C*, purple connections) were removed, resulting in an initialized MA network. Both network architectures were then trained separately but in exactly the same way (see below).

While each of the 12 different pairs of network instances had its own initial randomization of synaptic links and its own set of to-be-learned patterns, these features were identical to both pair members. Thus, there was some degree of 'between-subject' variability among the 12 network instances (because of randomly generated patterns and weight initializations for each pair), but there was parallelism with respect to these features between the two instances of each MA–HA pair. This ensured that the only difference between each MA–HA pair was in their long-distance connectivity—our variable of interest—while keeping all other factors identical. One may see this as the simulation of the same brain, once with human and once with monkey architecture, and thus as a “within-subject” manipulation.

### Training phase

The 14 different acoustic–articulatory patterns were generated for each pair of network instances including 17 specific cells in A1 and another 17 in M1, equaling 2.72% of the neurons in each respective 25 × 25 area. These neurons were thought to represent abstract articulatory and acoustic phonological features (including articulatory and acoustic phonological distinctive features and coarticulatory information) about spoken word forms. The selection of neurons was random and (again) identical between HA–MA pairs. When producing a spoken word form, specific articulatory movements yield acoustic signals, which, in turn, stimulate, with only minimal delay, the auditory system. To model this undeniable correlation of sensorimotor neuronal activity related to speech, which also receives support from recent electrocorticography studies (Cheung et al., 2016; Leonard et al., 2016), stimulus patterns were presented to the sensory and motor areas A1 and M1 networks. By “presenting a stimulus pattern,” we mean that its 2 × 17 cells were activated together for 16 time steps. We wanted to avoid any possibly contaminating activity related to the previously presented stimulus pattern, and hence an interstimulus interval (ISI) followed each stimulus presentation. This ISI lasted for at least 30 time steps, until network activity had returned to the baseline value. During these ISIs the only input to the network was uniform white noise, simulating the spontaneous baseline neuronal firing observed in real neurons. Note that all parts of the network were subjected to the same amount of noise. The trial-to-trial presentation sequence of the different patterns was random. Hebbian learning was effective throughout learning trials, both during stimulus presentation and ISIs.

After stimulation to M1 and A1, activation spread throughout the model areas. As a consequence of activation spreading and the resultant coactivation of neurons across the network, associative learning led to the formation of circuits interlinking the articulatory and auditory patterns, as documented in several previous studies (Garagnani et al., 2008; Garagnani and Pulvermüller, 2013, 2016; Pulvermüller and Garagnani, 2014; Tomasello et al., 2016). Due to sensorimotor activation, neural activity was present in specific neurons in A1 and M1, which partly activated further neural elements connected to these stimulated ones. Correlated activity and Hebbian learning mechanisms led to synaptic strengthening so that, eventually, sensorimotor stimulation led to increasingly stronger activation spreading to specific neuron sets throughout the network, which finally led to the formation of a distributed long-term memory (LTM) trace, or cell assembly (CA; for a detailed description and analyses of cell assembly formation in this type of network, see Garagnani et al., 2008, 2009; Pulvermüller and Garagnani, 2014).

### Testing phase

The functionality of the circuits developing in the HA and MA was then compared in the testing phase, where all previously learned auditory patterns were presented once again, in random order. Auditory stimulation (without articulatory pattern stimulation) was chosen to simulate speech perception. Stimulation was for two time steps; network responses were recorded during stimulation and the 30 subsequent time steps (i.e., 32 time steps in total).

### Data analysis

#### 

##### Structural network properties: cell assembly sizes.

To assess whether articulatory–acoustic learning led to cell assembly formation, the presence and sizes of these circuits were assessed in each network instance. To identify the neurons forming cell assemblies across the different network areas, the activity of all 3750 excitatory network cells was monitored in response to one specific stimulation pattern. For each area, we calculated the maximum firing rate occurring across all 625 excitatory cells of a given area at any time during the 30 time steps following sensory stimulation. A cell was considered a member of a given cell assembly if and only if at any time step its firing rate reached at least 50% of the firing rate of the maximally responsive cell in the given area at that time step (provided that such maximum firing rate was at least 0.2). These procedures and thresholds were chosen on the basis of simulation results obtained with the present and previous networks (Garagnani et al., 2008, 2009).

##### Dynamics of network activation.

We also analyzed neural dynamics within each area separately in response to learned patterns. To quantify differences in activation dynamics, we first calculated, for each area, the time point at which the firing rate was highest (*T*_max_). This value was then used to quantify the area-specific duration of sustained activity (which we interpret as a measure of verbal working memory; Fuster and Bressler, 2012), defined as the length of the interval (in simulation time steps) during which activity in an area remained significantly above the prestimulation average (≥2 SDs of the average firing rate in the 10 time steps immediately before stimulation). We refer to this quantity as the (area-specific) “sustained memory period” (SMP). For both T_max_ and SMP data, we conducted repeated-measures ANOVAs with factors model architecture (MA/HA) and area (six areas), both as within-subjects factor (see section Simulation procedures).

### Full model specification^1^

Each model area consists of two layers of 625 excitatory and 625 inhibitory cells (Fig. 1*E*). Each excitatory cell represents a cluster of cortical neurons (pyramidal cells), and the underlying inhibitory cell models the cluster of inhibitory interneurons situated within the same cortical column (Wilson and Cowan, 1972; Eggert and Van Hemmen, 2000). The state of each cell *x* is uniquely defined by its membrane potential *V*(*x*, *t*), representing the average of the sum of all (excitatory and inhibitory) postsynaptic potentials acting upon neural pool (cluster) *x* at time *t*, and governed by the following equation (see Table 1 for the parameter values used):

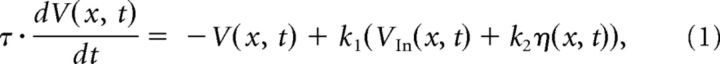
 where *V*_In_(*x*, *t*) is the net input to cell *x* at time *t* (sum of all IPSPs and EPSPs; inhibitory synapses are given a negative sign), τ is the time constant of the membrane, *k*_1_, *k*_2_ are scaling constants, and η(*x*, *t*) is a white noise process with uniform distribution over [−0.5, 0.5]. Time is in arbitrary units. Cells produce a graded response that represents the average firing rate of the neuronal cluster; in particular, the output (transformation function) of an excitatory cell *x* at time *t* is as follows:

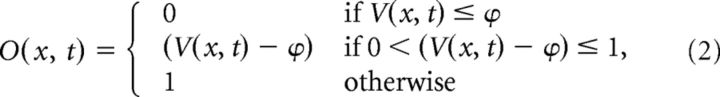
 where *O*(*x*, *t*) represents the average (graded) firing rate (number of action potentials per time unit) of cluster *x* at time *t*; it is a piecewise linear sigmoid function of the cell membrane potential *V*(*x*, *t*), clipped into the range [0, 1] and with slope 1 between the lower and upper thresholds ϕ and ϕ + 1. The output *O*(*x*, *t*) of an inhibitory cell is 0 if *V*(*x*, *t*) < 0, and *V*(*x*, *t*) otherwise. In excitatory cells, the value of the threshold ϕ in Equation 2 varies in time, tracking the recent mean activity of the cell so as to implement a simple version of neuronal adaptation (Kandel et al., 2000; higher activity leads to a higher threshold). More precisely, it is written as follows:

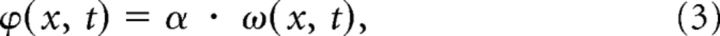
 where ω(*x*, *t*) is the time average of the recent output of the cell, and α is the “adaptation strength.”

**Table 1.** Parameter values used for the simulations

Equations	Parameters
1	Excitatory cells: τ = 2.5 (in simulation time steps) Inhibitory cells: τ = 5 (in simulation time steps)
	Scaling factor: *k*_1_ = 0.01
	Noise scaling factor (training phase): *k*_2_ = 15 √48
	Noise scaling factor (testing phase): *k*_2_ = 5 √48
	Global inhibition strength (training phase): *k_s_* = 95
	Global inhibition strength (testing phase): *k_s_* = 60
3	Adaptation: α = 0.026
4.1	Time constant for computing gliding average of cell activity: τ*_A_* = 15 (in simulation time steps)
4.2	τ*_S_* = 8
5	Postsynaptic potential thresholds for LTP: θ_+_ = 0.15
	Postsynaptic potential thresholds for LTD: θ_−_ = 0.15
	Presynaptic output activity required for any synaptic change: θ_pre_ = 0.05
	Learning rate: Δ*w* = 0.0007

For an excitatory cell *x*, the approximate time average ω(*x*, *t*) of its output *O*(*x*, *t*) is estimated by integrating the linear differential Equation 4.1 below with time constant τ*_A_*, assuming an initial average ω(*x*, 0) = 0, as follows:

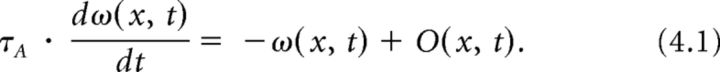
 Local (lateral) inhibitory connections (Fig. 1*E*) and area-specific inhibition are also implemented, realizing, respectively, local and global competition mechanisms (Duncan, 2006) and preventing activation from falling into nonphysiological states (Braitenberg and Schüz, 1998). More formally, in Equation 1 the input *V*_In_(*x*, *t*) to each excitatory cell of the same area includes an area-specific (“global”) inhibition term *k_S_* · ω*_S_*(*x*, *t*), which is subtracted from the total sum of the IPSP and EPSP postsynaptic potentials *V*_In_ in input to the cell, with ω_S_(*x*, *t*) defined as follows:

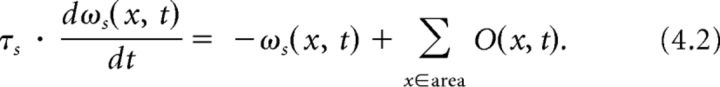
 The low-pass dynamics of the cells (Eqs. 1, 2, 4.1, 4.2) are integrated using the Euler scheme with step size Δ*t*, where Δ*t* = 0.5 (in arbitrary time units).

Excitatory links within and between (possibly nonadjacent) model areas are random and limited to a local (topographic) neighborhood; weights are initialized at random, in the range [0, 0.1]. The probability of a synapse to be created between any two cells falls off with their distance (Braitenberg and Schüz, 1998) according to a Gaussian function clipped to 0 outside the chosen neighborhood (a square of size *n* = 19 for excitatory cell projections and *n* = 5 for inhibitory cell projections). This produces a sparse, patchy, and topographic connectivity, as typically found in the mammalian cortex (Amir et al., 1993; Kaas, 1997; Braitenberg and Schüz, 1998; Douglas and Martin, 2004).

The Hebbian learning mechanism implemented simulates well documented synaptic plasticity phenomena of LTP and LTD, which are believed to play a key role in experience-dependent plasticity, memory, and learning (Rioult-Pedotti et al., 2000; Malenka and Bear, 2004). In particular, the learning rule is an implementation of the Artola–Bröcher–Singer model of LTP/LTD (Artola et al., 1990; Artola and Singer, 1993). In the model, we discretized the continuous range of possible synaptic efficacy changes into two possible levels, +Δ*w* and −Δ*w* (with Δ*w* ≪ 1 and fixed). We defined as “active” any link from an excitatory cell *x* such that the output *O*(*x*,*t*) of cell *x* at time *t* is larger than θ_pre_, where θ_pre_ ∈ ]0, 1] is an arbitrary threshold representing the minimum level of presynaptic activity required for LTP (or LTD) to occur. Thus, given any two cells *x* and *y* connected by a synaptic link with weight *w_t_*(*x*, *y*), the new weight *w_t_*_+1_(*x*, *y*) is calculated as follows:

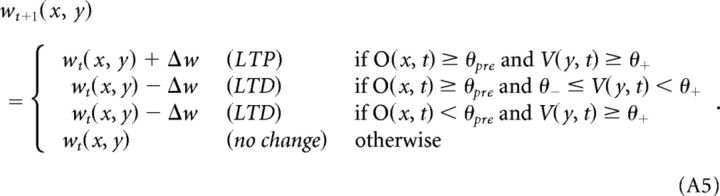


## Results

### Cell assembly sizes

**Figure 2.**
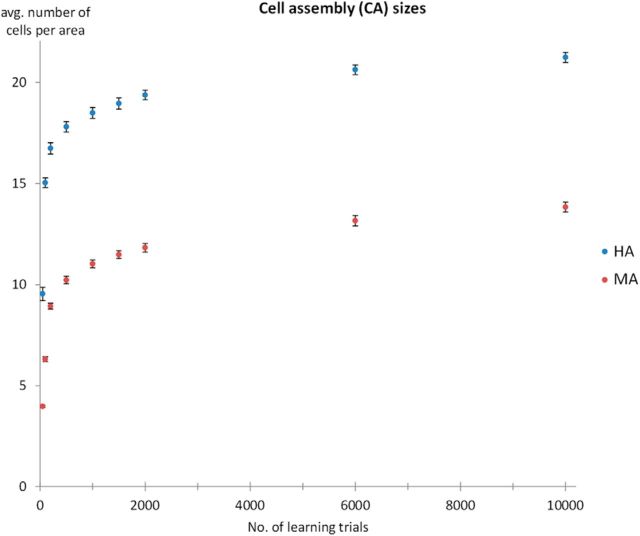
Cell assembly sizes (number of cells in CA) as a function of the number of learning trials. Data are presented separately for the MA (in red) and the HA (in blue). Each data point represents the average of 12 network instances with 14 patterns per network (*N* = 168). Error bars show SEM after removing between-network variance (Morey, 2008). Note the asymptotic behavior of both architectures with an increasing number of learning trials.

Before analyzing the dynamics of network activation, we computed the resulting cell assembly sizes obtained after representing the sensory part of a previously learned pattern to the model again (for cell assembly definition, see Materials and Methods). We computed CA sizes for 50, 100, 200, 500, 1000, 1500, 2000, 6000, and 10,000 learning trials. The resulting CA sizes are shown in Figure 2. CA sizes were always larger for the HA than the MA (all *p* < 0.001), regardless of the number of learning trials (presentations per pattern). During the first 1000 presentations, the number of CA cells grew at a very fast rate (relative ratios of CA sizes at 1000 versus 50 presentations were 1.82 for HA and 2.87 for MA). Growth rate fell off after 1000 presentations for both types of architectures (relative ratios of CA size ratios at 2000 versus 1000 presentations were 1.04 for HA networks and 1.06 for MA networks). These observations were supported by Tukey's HSD tests, which confirmed that CA sizes differed between 50 and 1000 presentations for both architectures (both *p* < 0.001), but did not significantly change between 1000 and 2000 presentations for either HA or MA networks (HA: *p* = 0.17; MA: *p* = 0.14). In addition, we approximated the derivative of the CA size changes at 500 and 1000 learning trials and found that at 500 time steps the size increase per additional learning trial (i.e., CA growth rate) was larger for MA (0.3 cells/100 learning trials) than HA (0.2 cells/100 learning trials). However, at 1000 time steps, this growth rate was 0.1 cells/100 learning trials for both architectures. Hence, we assume that at 1000 learning trials both networks had become relatively saturated with respect to learning, such that additional learning trials produced very small increases in CA sizes. We therefore focused further analyses on networks trained to 1000 learning trials.

### Dynamics of network activation

**Figure 3.**
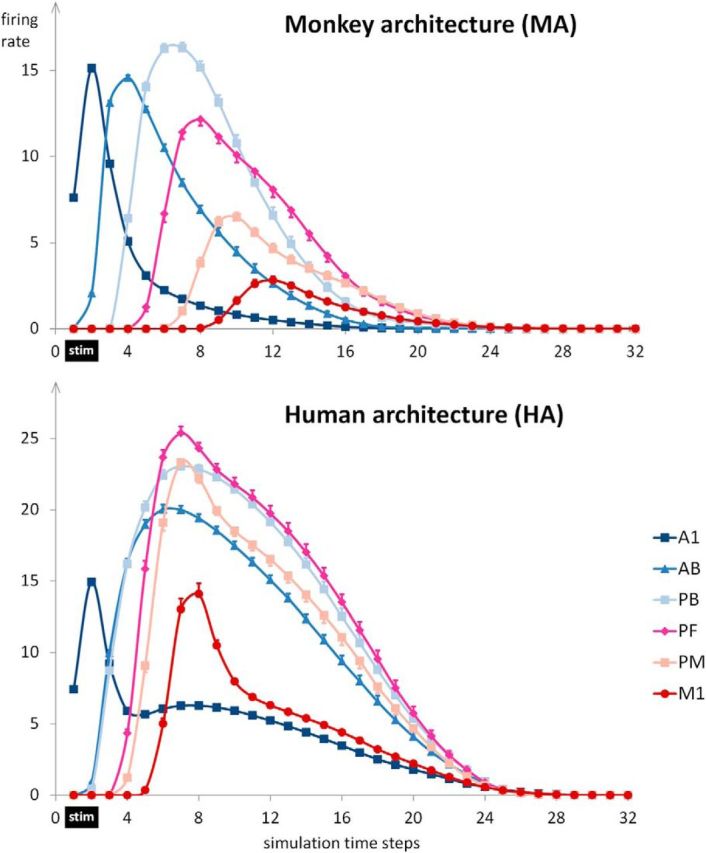
Dynamics of network activation after sensory stimulation. The panels show the sum of firing rates after presenting the sensory components of previously learned patterns to A1. Stimulation was for the first two time steps (marked by a black bar, “stim”), and, following this, firing rates were recorded for 30 time steps. As the sum of firing rates is shown, this measure reflects the total amount of activity in an area rather than average firing rate per cell. Each data point represents the average of 12 network instances with 14 patterns per network (*N* = 168). Error bars show SEM after removing between-network variance (Morey, 2008).

Figure 3 shows network dynamics (sum of firing rates as a function of simulation time step) induced by presentation of the sensory part of a previously learned word pattern to area A1 in the MA (top) and HA (bottom). Inspection of these plots reveals three qualitative differences in the dynamics of activation: (1) overall sum of firing rates are higher for the HA than for the MA (in part reflecting larger CA sizes; see Fig. 2); (2) activation is parallel for the HA, with areas AB/PB and PF/PM activating nearly simultaneously; in contrast, in the MA, activation spreads in a serial manner throughout the six areas; and (3) activation persists for a much longer time in the HA than in the MA.

**Figure 4.**
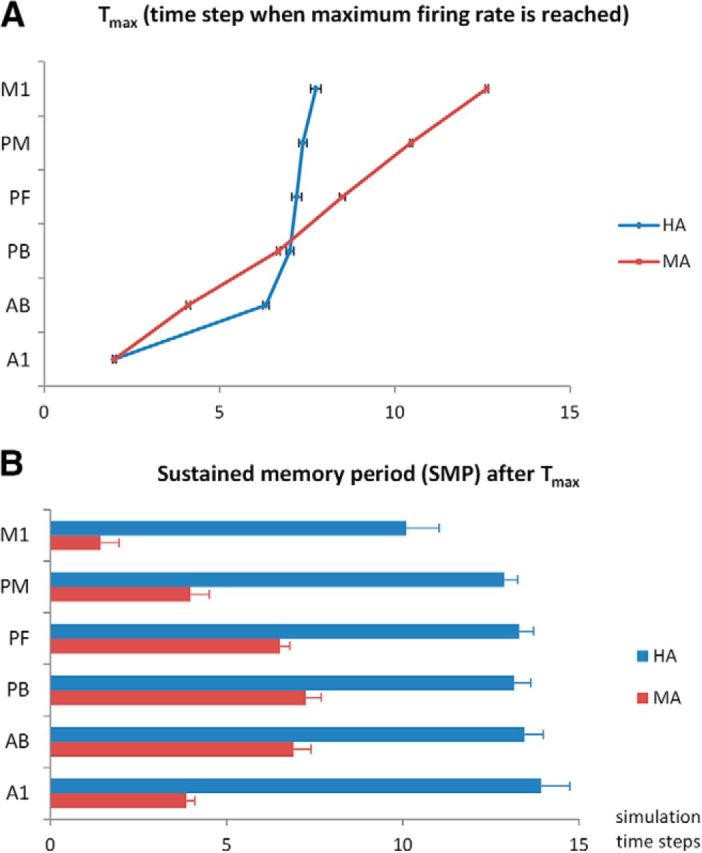
Quantitative analyses of the dynamics of network activation in the MA (in red) and the HA (in blue). ***A***, Time step when the maximum firing rate is reached, *T*_max_ (within the 30 poststimulation time steps only). Note the serial activation of the MA and the nearly simultaneous “ignition” effect of all areas except A1/AB in the HA. ***B***, SMP, defined as the duration (in time steps) starting from *T*_max_ during which the firing rate remained at ≥2 SDs of the average firing rate of the prestimulation phase. Note the significantly larger SMP values for the HA in all areas. Each data point represents the average of 12 network instances with 14 patterns per network (*N* = 168). Error bars show SEM after removing between-network variance (Morey, 2008). Both ***A*** and ***B*** are calculated based on the same data depicted in Figure 3 (prestimulation baseline period not depicted).

Note that the modeling results of serial versus parallel activation seem to match recent experimental results. Whereas in the auditory system of macaques, “latencies [of auditory-evoked activity sometimes] increase with increasing hierarchical region” (Camalier et al., 2012), a feature that Camalier et al. see as partly “consistent with [serial] anatomical predictions,” recordings from humans have been found to be “not supportive of a strict serial model envisioning principal flow of information” along the A1 to parabelt pathway (Nourski et al., 2014) but were supportive of largely parallel auditory area activation instead. This contrast, although coming from methodologically very different studies and only reflecting some aspects of extremely rich datasets, seems consistent with the tendencies toward serial versus parallel processing implicated by our MA and HA models, respectively (Figs. 3, 4*A*).

To investigate aspects 2 and 3 mentioned above (i.e., the seriality and persistence of activation quantitatively), we used the following measures (separately for each area and network type): the time step at which the maximum firing rate in a given area was reached, *T*_max_ (Fig. 4*A*) and the SMP (see Materials and Methods; Fig. 4*B*).

We also conducted separate ANOVAs on these two measures. For *T*_max_ data, the ANOVA revealed significant main effects of type (*F*_(1,11)_ = 446, *p* < 0.001) and area [*F*_(5,55)_ = 1878; Greenhouse-Geisser epsilon (GGe) = 0.52; *p* [GGe] < 0.001] and a significant interaction of type × area (*F*_(5,55)_ = 466; GGe = 0.49; *p* [GGe] < 0.001).

We conducted *post hoc* Tukey's HSD tests comparing, separately for HA and MA, *T*_max_ for adjacent areas. For the MA, all pairwise comparisons between adjacent areas were significant (*p* < 0.001), confirming the seriality of activation of adjacent areas. In contrast, for the HA, comparisons were not significant between adjacent areas PB and PF (mean difference = 0.19 time steps; *p* = 0.98), between PF and PM (mean difference = 0.18 time steps; *p* = 0.99), between PM and M1 (mean difference = 0.37; *p* = 0.37), or even between the nonadjacent areas PB and PM (mean difference = 0.37; *p* = 0.37). All other comparisons between adjacent areas (A1 and AB, AB and PB) for the human architecture were significant (all *p* < 0.001). This indicates that in the HA, activation initially spread serially from A1 via AB to PB, at which point the remaining areas of PF, PM, and M1 activated nearly simultaneously (Fig. 4*A*).

For SMP data, the ANOVA revealed significant main effects of type (*F*_(1,11)_ = 1388, *p* < 0.001) and area (*F*_(5,55)_ = 186; GGe = 0.63; *p* [GGe] < 0.001) and a significant interaction of type × area (*F*_(5,55)_ = 62; GGe = 0.54; *p* [GGe] < 0.001).

We conducted *post hoc* Tukey's HSD tests on the SMP data, comparing HA and MA at for each area separately. These comparisons showed that the SMP was significantly larger for the HA than for the MA in all six areas (all *p* < 0.001).

### Repetition of analyses comparing HA 1000 to MA 10,000

Although, as described in the Results section for cell assembly sizes, after 1000 learning trials, both models were at comparable learning stages, we wanted to rule out the possibility that the MA is simply slower in learning but still able to achieve qualitatively similar results. Therefore, we tested whether all statistical analyses obtained by comparing the HA and MA still obtained even when comparing the HA after 1000 learning trials to the MA after 10,000 learning trials. The ANOVAs for *T*_max_ and SMP data provided the same significance level for all effects. Thus, even when giving the weaker architecture the benefit of a 10-fold increase in learning trials, fundamental differences remained between the dynamics of network activation, and, hence, we can rule this out as a confound.

## Discussion

We used a neural network model mimicking fronto-temporal perisylvian language areas, including primary sensorimotor, secondary, and multimodal brain areas, to simulate word learning and examine network responses to the sensory (“auditory”) component of a previously learned pattern, akin to perceiving a familiar spoken word. Crucially, we compared the performances of two types of architectures, MA (monkey architecture) and HA (human architecture), implementing differences in the connectivity of perisylvian areas suggested by DTI/DWI tractography in monkeys/apes and humans. Our results showed the following advantages of the HA over the MA: (1) larger overall size of cell assemblies or action–perception circuits (APCs; Fig. 2), and thus stronger and more robust circuit activation (Fig. 3); (2) parallel rather than serial activation reflecting cell assembly ignition (Figs. 3, 4*A*); and (3) long-lasting activity in the network, reflecting cell assembly reverberation, and hence, emergence of VWM only in the HA (Figs. 3 and 4*B*).

Crucially, the disadvantages of the MA could not be compensated by longer training (up to 10,000 learning trials; see Results).

### What are the linguistic implications of the observed functional changes?

These results suggest that a change in neuroanatomical connectivity structure emerging in primate evolution underlies the build-up of a functionally robust lexicon of neuronal memory traces for multimodal articulatory-auditory patterns. Although the present simulations did not implement semantics, the emergent neuronal assemblies with long-lasting reverberating activity can be seen as a prerequisite for building a cortical lexicon of meaningful words. As complementary simulation studies show, such semantic learning is possible based on the same mechanisms of correlation mapping as those functional in the current model (Garagnani and Pulvermüller, 2016; Tomasello et al., 2016). In contrast to the large and fast-activating cell assemblies in the HA, the smaller and functionally sluggish circuits in the MA activated in a serial fashion, area by area, and, despite this prolonged activation process, there was little-to-no reverberatory activity (SMP; Fig. 4*B*). In contrast, the HA yielded longer-persisting activity in its APCs, which we interpret as signifying verbal (phonological) working memory (Fuster and Bressler, 2012; Zipser et al., 1993).

The evolutionary change in neuroanatomical structure may provide a partial explanation for why nonhuman primates have extremely weak auditory working memory, not only compared to humans but also compared to primates' working memory abilities in other sensory modalities (Fritz et al., 2005; Scott et al., 2012, 2014), and why, even after extensive training, nonhuman primates achieve vocabularies of only a fraction of those seen in humans (Savage-Rumbaugh et al., 1993; Call and Tomasello, 2007).

### The functional relevance of the motor system for VWM

It is widely agreed that a main function of the arcuate fasciculus is to map acoustic to articulatory representations. In our models, when presenting learned auditory patterns to A1, sustained activation in motor areas (M1 and PM)—those areas most distant from the sensory input—was observed only in the HA, and it occurred earlier than in the MA (Figs. 3, 4*B*). This motor activity in our model can be viewed as reflecting (subvocal) articulation or rehearsal processes in a “phonological loop” (Baddeley, 2003). Our results thus support the idea that VWM is not subserved by any dedicated module but, rather, consists in reverberating activity between frontal and temporoparietal areas, in line with patient, neuroimaging, and transcranial magnetic stimulation (TMS) evidence demonstrating the importance of speech perception and production areas in VWM (Belleville et al., 1992; Paulesu et al., 1993; Wilson, 2001; Buchsbaum et al., 2005; Jacquemot and Scott, 2006; Romero et al., 2006; Buchsbaum and D'Esposito, 2008; Acheson et al., 2011; Liao et al., 2014). Hence, an obvious explanation of why AF strength is important for VWM is that the AF enables quick and efficient sensory-to-motor coupling along the dorsal stream for retrieving word form representations when listening and thereby activates bidirectional auditory-to-motor and motor-to-auditory loops for activity maintenance in reverberating working memory circuits (Pulvermüller and Garagnani, 2014).

Individual differences in the degree of motor systems recruitment during speech perception could also contribute to differential working memory abilities. Correlations between verbal working memory performance and speech motor system activations during speech perception have been demonstrated, both in fMRI (Szenkovits et al., 2012) and using motor-evoked potentials (Murakami et al., 2015). Hence, one can speculate that higher verbal working memory abilities are driven by stronger motor systems recruitment, although the existing studies do not allow definite conclusions about the causality of this relationship.

We note that one prediction emerging from the present account is that the producibility of incoming auditory stimuli should influence working memory. Producibility of speech sounds influences the activation of motor areas (Wilson and Iacoboni, 2006) and auditory-to-motor connectivity (Londei et al., 2010) during perception. If this motor activation is also functionally relevant for verbal working memory, then producibility should similarly influence the learning of novel word forms. Indeed, producibility has been shown to influence recognition accuracy in word learning (Schulze et al., 2012). Furthermore, neurophysiological memory traces for newly learned word forms differ depending on whether they exhibit native-like—and hence pronounceable—phonology (Kimppa et al., 2015) and also depending on whether they are actually articulated during learning (Pulvermüller et al., 2012).

### The relation between working memory and language learning

Just like large vocabularies, VWM is a unique feature of humans, and even across human individuals there seem to be intrinsic relationships between VWM and language-learning abilities (Baddeley et al., 1988; Baddeley, 1993; Papagno and Vallar, 1995). Furthermore, speech production deficits can lead to reduced vocabulary size, presumably due to impairments in overt or covert repetition of novel pseudowords (Bishop et al., 1990). Finally, the perisylvian areas implicated in articulatory rehearsal have been shown to also be important for word recognition memory by fMRI (Wagner et al., 1998; Davachi et al., 2001; Clark and Wagner, 2003; Paulesu et al., 2009) and non-invasive brain stimulation experiments (Karabanov et al., 2015; Savill et al., 2015).

In essence, current theory and data strongly support that word learning in humans requires and relies on VWM. Human (anterior and posterior) perisylvian cortex provides the substrate for VWM, and the perisylvian dorsal connection by way of the AF plays a crucial role in word learning (López-Barroso et al., 2013), likely in concert with the extreme capsule (López-Barroso et al., 2011). This is not to say that perisylvian connectivity is the only relevant factor, as other structures, notably the hippocampus (Breitenstein et al., 2005; Sederberg et al., 2007) and the amygdala (Ripollés et al., 2014), play important complementary roles in word learning.

### What are the critical variables and benefits of the evolutionary network topological change?

Although there is agreement that the human AF is important for language (Wernicke, 1874; Rilling et al., 2008) and experimental evidence demonstrates its importance for verbal working memory (Benson et al., 1973; Damasio and Damasio, 1980; Catani et al., 2007; Rauschecker et al., 2009; Buchsbaum et al., 2011), the precise reason and underlying cortical mechanisms for these structure–function relationships had long remained unclear. Carefully controlled comparison of neural architectures may help here, as these can be exactly parallelized so that any functional difference between architectural “twins” can be uniquely attributed to the one and only manipulated structural feature. In our present case, this specific feature was the implementation of strong corticocortical “jumping link” connections, which, as suggested by comparative neuroanatomical studies using DTI/DWI tractography (see Introduction), may constitute an important structural difference between human and nonhuman primate brains. These connections provide “shortcuts” in the auditory-articulatory pathway in left perisylvian cortex, leading to shorter sensorimotor path length. In general, path length is an important feature of cortical neuroanatomy, which can be used to characterize functionally relevant differences (Kaiser and Hilgetag, 2006; van den Heuvel and Sporns, 2013). Furthermore, as more connections were present in the HA, multiple parallel links became available for projecting acoustic and articulatory phonological information onto each other. Rapid activation flow between articulatory and auditory regions appears necessary for building “actively” reverberating loops, providing the rehearsal mechanism in human verbal working memory, and it is precisely this active memory component that nonhuman primates lack (Scott and Mishkin, 2016). Hence, we propose that these two features taken together—the more numerous connections and their shorter path lengths—are the crucial variables determining the more robust “word representations” and the emergence of verbal (phonological) working memory in humans. Short sensorimotor path length may offer a mechanism not only for verbal working memory, but also for the coupling of auditory and motor information related to speech (for a related computational model, see Westermann and Miranda, 2004). This coupling could also explain why auditory-articulatory interactions are pervasive in speech perception and comprehension (Schomers and Pulvermüller, 2016; Skipper et al., 2017).

### Conclusions

Our results suggest that the AF plays a critical role in word learning because its rich connectivity in humans allows for efficient binding of auditory and articulatory information about speech into persistently active neuronal circuits carrying VWM functions. As VWM is necessary for acquiring a vast repertoire of meaningful words, such strongly reverberating circuits may be essential for explaining human language. We believe that the present comparative-neurocomputational research approach may open new and exciting pathways for explanatory evolutionary neuroscience.

## Footnotes

^1^This has been adapted from Garagnani and Pulvermüller (2013).
